# QT Independent Ventricular Tachycardia Induced by Arsenic Trioxide

**DOI:** 10.1155/2019/9870283

**Published:** 2019-10-13

**Authors:** Vicken Zeitjian, Carmel Moazez, Waqas Arslan, Mehrdad Saririan

**Affiliations:** ^1^Department of Cardiovascular Diseases, University of Texas San Antonio Health Science Center, 7703 Floyd Curl Dr. San Antonio, TX 78229, USA; ^2^Department of Internal Medicine, Creighton University Maricopa Medical Center, 2601 E. Roosevelt St. Phoenix, AZ 85006, USA; ^3^Department of Hematology and Oncology, Creighton University Maricopa Medical Center, 2601 E. Roosevelt St. Phoenix, AZ 85006, USA; ^4^Department of Cardiovascular Diseases, Creighton University Maricopa Medical Center, 2601 E. Roosevelt St. Phoenix, AZ 85006, USA

## Abstract

Arsenic trioxide (ATO) is commonly known to cause QT prolongation with resultant ventricular tachycardia (VT). VT, independent of QT prolongation, can be a complication of ATO. We present a 46-year-old female who received ATO and during her hospital course had intermittent nonsustained VT. All usual causes of VT were considered including reduced EF < 35%, ischemia, electrolyte abnormalities, medications, and genetic polymorphisms; however, no specific cause was found. After stopping therapy, the episodes of nonsustained VT ceased indicating that there is an association between ATO and VT.

## 1. Introduction

Arsenic trioxide (ATO) is commonly known to cause QT prolongation with resultant ventricular tachycardia (VT); however, it is the preferred treatment in combination with all-trans retinoic acid for acute promyeloctyic leukemia. VT, independent of QT prolongation, is a complication of ATO. Here, we present a case of nonsustained VT without QT prolongation due to ATO. This is the second paper exploring the association of nonsustained VT and ATO due to a mechanism different than prolonged QT.

## 2. Case

A 46-year-old Hispanic female presented with a chief complaint of generalized fatigue, vaginal bleeding, gingival bleeding, and nasal bleeding for 2 weeks. On exam, she was tachycardic and had conjunctival pallor. She had several areas of ecchymosis over her extremities and abdomen and scattered petechiae across the chest. Labs were significant for a hemoglobin of 7.7 g/dL, white blood cells of 3.3/*μ*L, platelets of 3 K/*μ*L, and blasts of 37%. Peripheral smear showed progranulocytes and auer rods. Fluorescent in situ hybridization was done on bone marrow showing promyelocytic leukemia-retinoic acid receptor alpha (PML-RARA) positivity indicating acute promyelocytic leukemia.

**Figure 1:**
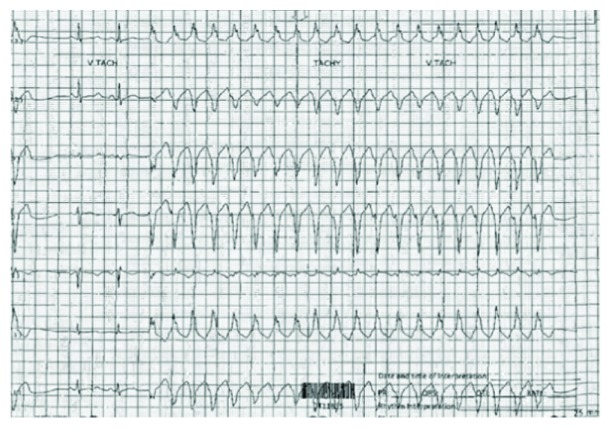
Nonsustained monomorphic ventricular tachycardia.

She was started on all-trans retinoic acid (ATRA) and ATO for intermediate risk APL with treatment length until remission or up to 60 days, whichever came first. A combination of ATRA and ATO was chosen based on their efficacy of obtaining remission compared to ATRA combined with standard chemotherapy. Initial electrocardiogram (EKG) showed sinus tachycardia with a heart rate of 110 and corrected QT (QTc) interval of 434 ms. On day 26 of ATO treatment, she complained of palpitations and began having episodes of asymptomatic nonsustained VT on telemetry (Figure 1). EKG was done which was unable to capture the VT. The rhythm was sinus with QTc of 447 ms. Potassium was 4 mg/dL and magnesium was 2.3 mg/dL. She had no family history of sudden cardiac death or related long QT sequelae. Baseline EKG had a normal QTc.

She had a transthoracic echocardiogram, which showed no structural heart disease and a normal ejection fraction. We considered the option of discontinuing ATO; however, our oncology consultants felt strongly about the need to continue combination ATRA and ATO because the patient had at least intermediate risk APL. She was started on nadolol 40 mg daily as off-label treatment given that ATO continuation was necessary for up to 60 days. We chose 40 mg as the starting dose, which is the minimum dose of nadolol with plans of uptitrating if VT was not suppressed. Since there were no further reoccurrences of nonsustained VT, we did not have to increase the dose. Episodes of nonsustained VT were decreased after starting nadolol. Nadolol was chosen as a once daily beta-blocker based on its efficacy of reducing ventricular ectopy and tachycarrhythmias. Fortunately, the patient was able to continue cancer treatment to completion. The patient completed 60 days of ATO treatment with weekly QTc monitoring and was continued on nadolol until completion of treatment.

## 3. Discussion

ATO has been known to cause VT due to related long QT. However, through this case and literature review, we propose that ATO is also associated with VT independent of prolonged QT. QT prolongation, VT, heart block, and sudden cardiac death have been reported with ATO use. Prolonged QT is the most common and occurs in 26-93% of cases. It commonly occurs after 1-5 weeks of therapy and usually returns to baseline after 8 weeks from the last infusion [1, 2].

In our case, all usual causes of VT were considered including reduced EF < 35%, ischemia, electrolyte abnormalities, medications, idiopathic VT, and genetic polymorphisms; however, no specific cause was found. No invasive studies were performed given that after discontinuation of ATO, the episodes of VT ceased leading us to believe that there is an association.

Ducas et al. presented the first case of VT during ATO treatment independent of QTc. Their investigation for reversible causes of VT was also negative leading the authors to believe ATO as a cause of VT [3]. Arsenic cardiotoxicity is a known pathology well described in the National Comprehensive Cancer Network (NCCN). Per NCCN recommendations, a 12 lead EKG, creatinine, and electrolytes including potassium, magnesium, and calcium are required prior to commencement of therapy. During treatment, weekly EKG and electrolytes should be monitored. Target levels include a QTc of <460 ms (in females), potassium greater than 4 mmol/L, and magnesium greater than 0.74 mmol/L [3, 4].

Current ESC guidelines recommend discontinuing or lowering the dosing of ATO in patients with prolonged QTc > 500 ms to prevent torsade de pointes. Also, providers should perform weekly EKGs and check electrolytes while patients are receiving ATO. Our patient developed VT independent of prolonged QTc, and therefore, we opted to not discontinue or reduce her treatment, as she did not have a prolonged QTc [5].

ATO is known to uncouple cardiac mitochondrial oxidative phosphorylation due to competing with inorganic phosphate for ATP formation. Intracellular ATP will become depleted, and the cardiac I_K-ATP_ will be activated as a compensatory mechanism. These channels increase potassium conduction and stabilize the resting membrane potential. As a result, there is a shorter action potential with decreased calcium influx and preservation of intracellular energy. ATO is also a potent blocker of I_Kr_ and I_Ks_ which activate delayed rectified potassium channels [6]. Delayed rectified potassium channels are responsible for ventricular repolarization and stabilization of membrane potential. With disruption of the normal function of these channels and multiple potassium currents, electrical instability may result in malignant tachyarrhythmias [7].

We conclude that ATO may induce VT with and without association of QT prolongation. ATO's intrinsic properties affecting potassium ion channels are the likely cause of VT occurrence. After completing ATO, she has been free of ventricular tachycardia for the last 6 months.

## 4. Conclusion

We conclude that ATO may induce VT independent of QTc prolongation. Previous studies in patients on ATO have shown VT to be dependent on QTc prolongation. Our paper is the 2nd reported case documenting the association between VT and ATO independent of the QTc interval. This clinical observation supports close telemetry monitoring in patients who report palpitations as VT may be the underlying etiology. The mechanism of VT remains unclear, and therefore, further studies at an ionic level may help investigate VT and ATO association.
